# Treatment of Obstinate Ulcers

**Published:** 1853-02

**Authors:** J. Tart


From the Provincial Med. and Surg. Journal.

*Treatment of Obstinate Ulcers by the Internal use of Tincture of Cantharides.* By J. Tart, Esq.

In a case of extensive ulceration in a broken constitution, after the failure of various plans of treatment, Mr. Tart gave ten drops of tincture of cantharides three times a day, with marked benefit. In three days from the commencement, the sores began to contract, healthy lymph appeared round the edges, and vivid granulations started up. In a fortnight, the ulcers were quite healed. On this case, the author remarks:—

"Such was the progress and issue of a case, that had baffled every previous treatment employed. It affords one of many examples I could bring forward, of the great utility of cantharides in indolent ulceration, dependent either upon atony of the engaged parts, or system generally.

"In 1845, while resident in Burmah, my attention was directed to the treatment of the ulcers met with in that country, and which had long been found difficult to heal by different medical gentlemen stationed upon that coast. I drew up a paper, exhibiting the appearances presented by the different ulcers, and the states of constitutional derangement with which they were identified, and in which I had employed the tincture of cantharides with marked success. The paper alluded to, backed by several cases treated by different medical friends, was forwarded to the Madras Medical Board, who ordered it to be circulated throughout the medical service of the Madras army.

"A few extracts from the paper here referred to will show the characters of the ulcers where I found the tincture of cantharides useful:—

"1st. Where the granulations were exuberant, but pale, weak, and flabby.

"2d. Where there was deficiency, or total absence of granulations, the ulcers being deep and scooped out, with raised and indurated edges.

"3d. Where the granulations were not defective, but cicatrizing irregularly, sometimes in the centre, at other times on one side, the lymph which was thrown out and organised one day being absorbed the next."

